# Acute exacerbation predicting poor outcomes in idiopathic interstitial pneumonia and advanced lung cancer patients undergoing cytotoxic chemotherapy

**DOI:** 10.1038/s41598-024-60833-w

**Published:** 2024-05-03

**Authors:** Atsushi Miyamoto, Hirofumi Michimae, Yasuharu Nakahara, Shinobu Akagawa, Kazuhiko Nakagawa, Yuji Minegishi, Takashi Ogura, Shigeto Hontsu, Hiroshi Date, Kazuhisa Takahashi, Sakae Homma, Kazuma Kishi, Investigators Group for Lung Cancer and IIP, Y. Nakahara, K. Ohta, A. Gemma, Y. Nishizaka, T. Ogura, H. Kimura, K. Nishi, M. Nakamura, K. Yokomura, H. Taniguchi, K. Tomii, J. Shindo, K. Sato, Y. Taguchi, H. Takahashi, H. Takizawa, S. Homma, S. Nakamura, K. Yoshimura, K. Usui, K. Ichikado, A. Bessyo, H. Sugiyama, Y. Hasegawa, H. Nakamura, H. Sagara, K. Ube, F. Nomura, K. Kiura, F. Yoshiike, K. Takahashi, T. Kita, H. Sakai, M. Bando, T. Matsumoto, T. Inoue, T. Kijima, H. Mukae, N. Masuda, N. Matsumoto, F. Sakamaki, M. Kamimura, A. Takise, T. Kishaba, Y. Nishioka, K. Kashiwabara, A. Yamamoto, S. Fujiuchi, M. Shingyoji, M. Hanaoka, S. Tominaga, J. Kadota, T. Kasahara, M. Motegi, T. Harada, S. Ishikawa, T. Suda, Y. Tomizawa, R. Hayashi, M. Shinoda, M. Terada, Y. Jin, Y. Shikama, T. Kikuchi, K. Kido, A. Yokoyama, S. Fuke, H. Nagase, H. Tanaka, N. Hizawa, K. Miyazaki, S. Ikushima, N. Sakai, T. Hoshino, M. Mishima, H. Ohnishi, H. Imai, S. Nagashima, E. Kojima, S. Ohishi, Y. Ohe, S. Iwakami, M. Mineshita, Y. Komase, H. Harada, S. Imokawa, H. Watanabe, M. Ichiki, K. Kuwano, N. Takahashi, N. Chonabayashi, T. Hisada, M. Yoshida, K. Hirata, K. Watanabe, Y. Sugino, S. Yoshioka, H. Tomioka, M. Aoshima, Y. Sugimoto, M. Ichinose, S. Tamaki, M. Tsuchiya, H. Katayama, Y. Okochi, H. Tanaka, K. Ogata, T. Tsuburai, I. Honda

**Affiliations:** 1 Department of Respiratory Medicine, Respiratory Centre, Toranomon Hospital, 2-2-2 Toranomon Minato-ku, Tokyo, 105-8470 Japan; 2 School of Pharmacy, Department of Clinical Medicine (Biostatistics), Kitasato University, 5-9-1 Shirokane Minato-ku, Tokyo, 108-8642 Japan; 3 Department of Respiratory Medicine, National Hospital Organization, Himeji Medical Centre, 68 Hon-machi, Himeji-shi, Hyogo, 670-8520 Japan; 4 Department of Respiratory Medicine, National Hospital Organization, Tokyo National Hospital, 3-1-1 Takeoka, Kiyose-shi, Tokyo, 204-8585 Japan; 5 Department of Respiratory Medicine, Japanese Red Cross Osaka Hospital, 5-30 Fudegasakicho, Tennoji-ku, Osaka, 543-8555 Japan; 6 Department of Pulmonary Medicine and Oncology, Graduate School of Medicine, Nippon Medical School University, 1-1-5 Sendagi Bunkyo-ku, Tokyo, 113-8602 Japan; 7 Department of Respiratory Medicine, Kanagawa Cardiovascular and Respiratory Centre, 6-16-1 Tomioka-higashi Kanazawa-ku, Yokohama-shi, Kanagawa, 236-0051 Japan; 8 Department of Respiratory Medicine, Nara Medical University, 840 Shijo-cho, Kashihara, Nara 634-8521 Japan; 9 Department of Thoracic Surgery, Kyoto University Graduate School of Medicine, 54 Shogoin-Kawahara-cho, Sakyo-ku, Kyoto, 606-8507 Japan; 10 Department of Respiratory Medicine, Juntendo University Graduate School of Medicine, 2-1-1 Hongo, Bunkyo-ku, Tokyo, 113-8421 Japan; 11 Department of Pulmonary Medicine, Toho University School of Medicine, 5-21-16 Omori-nishi, Ota-ku, Tokyo, 143-8540 Japan; 12 Okinaka Memorial Institute for Medical Research, 2-2-2 Toranomon, Minato-ku, Tokyo, 105-8470 Japan; 13 Present Address: Department of Respiratory Medicine, Mitsui Memorial Hospital, Kanda-Izumi-cho 1, Chiyoda-ku, Tokyo, 101-8643 Japan; 14 Present Address: Department of Pulmonary Medicine, Toho University School of Medicine, 5-21-16 Omori-nishi, Ota-ku, Tokyo, 143-8540 Japan; 15 National Hospital Organization (NHO), Himeji Medical Centre, Himeji, Japan; 16 NHO Tokyo National Hospital, Tokyo, Japan; 17 Nippon Medical School University, Tokyo, Japan; 18 Japanese Red Cross Osaka Hospital, Osaka, Japan; 19 Kanagawa Cardiovascular and Respiratory Centre, Yokohama, Japan; 20 Nara Medical University, Kashihara, Japan; 21 Ishikawa Prefectural Central Hospital, Kanazawa, Japan; 22 Tokyo Saiseikai Central Hospital, Tokyo, Japan; 23 Seirei Mikatahara General Hospital, Hamamatsu, Japan; 24 Tosei General Hospital, Seto, Japan; 25 Kobe City Medical Centre General Hospital, Kobe, Japan; 26 Ogaki Municipal Hospital, Ogaki, Japan; 27 Nagaoka Red Cross Hospital, Nagaoka, Japan; 28 Tenri Hospital, Tenri, Japan; 29 Sapporo Medical University, Sapporo, Japan; 30 Kyorin University, Mitaka, Japan; 31 Toho University Omori Medical Centre, Tokyo, Japan; 32 Funabashi Municipal Medical Centre, Funabashi, Japan; 33 Mitsui Memorial Hospital, Tokyo, Japan; 34 NTT Medical Centre Tokyo, Tokyo, Japan; 35 Saiseikai Kumamoto Hospital, Kumamoto, Japan; 36 Okayama Red Cross General Hospital, Okayama, Japan; 37 Centre Hospital of the National Centre for Global Health and Medicine, Tokyo, Japan; 38 Osaka Saiseikai Nakatsu Hospital, Osaka, Japan; 39 Seirei Hamamatsu General Hospital, Hamamatsu, Japan; 40 Showa University, Tokyo, Japan; 41 Iwate Prefectural Central Hospital, Morioka, Japan; 42 Japanese Red Cross Nagoya Daiichi Hospital, Nagoya, Japan; 43 Okayama University, Okayama, Japan; 44 Nagano Municipal Hospital, Nagano, Japan; 45 Juntendo University, Tokyo, Japan; 46 National Hospital Organization, Kanazawa Medical Centre, Kanazawa, Japan; 47 Saitama Cancer Centre, Saitama, Japan; 48 Jichi Medical University, Shimotsuke, Japan; 49 Tomishiro Central Hospital, Tomigusuku, Japan; 50 Sano Kosei General Hospital, Sano, Japan; 51 Osaka University, Osaka, Japan; 52 University of Occupational and Environmental Health, Kitakyushu, Japan; 53 Kitasato University, Tokyo, Japan; 54 University of Miyazaki, Miyazaki, Japan; 55 Tokai University Hachioji Hospital, Hachioji, Japan; 56 NHO Disaster Medical Centre, Tokyo, Japan; 57 Japanese Red Cross Maebashi Hospital, Maebashi, Japan; 58 Okinawa Chubu Hospital, Uruma, Japan; 59 Tokushima University, Tokushima, Japan; 60 Kumamoto Regional Medical Centre, Kumamoto, Japan; 61 Takamatsu Red Cross Hospital, Takamatsu, Japan; 62 NHO Asahikawa Medical Centre, Asahikawa, Japan; 63 Chiba Cancer Countermeasure, Chiba, Japan; 64 Shinshu University, Matsumoto, Japan; 65 Juntendo University, Urayasu Hospital, Urayasu, Japan; 66 Faculty of Medicinem, Oita University, Oita, Japan; 67 Kanazawa University, Kanazawa, Japan; 68 National Hospital Organization, Takasaki General Medical Centre, Takasaki, Japan; 69 Japan Community Health Care Organization (JCHO), Hokkaido Hospital, Sapporo, Japan; 70 NHO Chiba-East-Hospital, Chiba, Japan; 71 Hamamatsu University, Hamamatsu, Japan; 72 NHO Shibukawa Medical Centre, Shibukawa, Japan; 73 Toyama University, Toyama, Japan; 74 Yokohama City University Medical Centre, Yokohama, Japan; 75 Saiseikai Niigata Hospital, Niigata, Japan; 76 Hiratsuka Kyosai Hospital, Hiratsuka, Japan; 77 Showa University, Northern Yokohama Hospital, Yokohama, Japan; 78 Niigata University, Medical and Dental Hospital, Niigata, Japan; 79 Juntendo University, Nerima Hospital, Nerima, Japan; 80 Kochi Medical School, Nankoku, Japan; 81 KKR Sapporo Medical Centre, Sapporo, Japan; 82 Teikyo University, Itabashi City, Japan; 83 Niigata Cancer Centre Hospital, Niigata, Japan; 84 University of Tsukuba, Tsukuba, Japan; 85 Ryugasaki Saiseikai Hospital, Ryugasaki, Japan; 86 Japanese Red Cross Medical Centre, Shibuya City, Japan; 87 Japanese Red Cross Otsu Hospital, Otsu, Japan; 88 Kurume University, Kurume, Japan; 89 Kyoto University, Kyoto, Japan; 90 Akashi Medical Centre, Akashi, Japan; 91 Gunma Prefectural Cancer Centre, Ota, Japan; 92 NHO Nagasaki Medical Centre, Ōmura, Japan; 93 Komaki City Hospital, Komaki, Japan; 94 NHO Ibarakihigashi National Hospital, Naka, Japan; 95 National Cancer Centre Hospital, Chuo City, Japan; 96 Juntendo University, Shizuoka Hospital, Izunokuni, Japan; 97 St. Marianna University School of Medicine, Kawasaki, Japan; 98 St. Marianna University School of Medicine, Yokohama Seibu Hospital, Yokohama, Japan; 99 Yao Tokushukai General Hospital, Osaka, Japan; 100 Iwata City Hospital, Iwata, Japan; 101 Saka General Hospital, Shiogama, Japan; 102 NHO Kyushu Medical Centre, Fukuoka, Japan; 103 The Jikei University School of Medicine, Minato City, Japan; 104 Itabashi Hospital, Nihon University, Itabashi City, Japan; 105 St. Luke’s International Hospital, Tokyo, Japan; 106 Gunma University, Maebashi, Japan; 107 NHO Fukuoka Hospital, Fukuoka, Japan; 108 Osaka City University School of Medicine, Osaka, Japan; 109 Fukuoka University, Fukuoka, Japan; 110 TOYOTA Memorial Hospital, Toyota, Japan; 111 Nagasaki Harbor Medical Centre, Nagasaki, Japan; 112 Kobe City Hospital Organization, Kobe City Medical Centre West Hospital, Kobe, Japan; 113 Kameda Medical Centre, Kamogawa, Japan; 114 Tottori Prefectural Central Hospital, Tottori, Japan; 115 Tohoku University School of Medicine, Sendai, Japan; 116 NHO Nara Medical Centre, Nara, Japan; 117 Rakuwakai Otowa Hospital, Kyoto, Japan; 118 Ehime University School of Medicine, Toon, Japan; 119 JCHO Tokyo Yamate Medical Centre, Shinjuku City, Japan; 120 Senju Hospital, Sasebo, Japan; 121 Shindenbaru Seibo Hospital, Yukuhashi, Japan; 122 NHO Sagamihara National Hospital, Sagamihara, Japan; 123 Omuta Tenryo Hospital, Omuta, Japan

**Keywords:** Medical research, Oncology

## Abstract

Effective treatment for advanced lung cancer and idiopathic interstitial pneumonia (IIP) remains an unmet medical need. The relationship between chemotherapy’s effectiveness in advanced lung cancer and the risk of acute exacerbation of IIP is poorly investigated. There is limited evidence that patients who experience an acute exacerbation of IIPs during cytotoxic chemotherapy have poorer outcomes than those who do not. Among 1004 patients with advanced lung cancer and IIPs enrolled in our published multi-centre retrospective study from 110 Japanese institutions, 708 patients (male: female, 645:63; mean age, 70.4) received first-line chemotherapy. The occurrence of chemotherapy-triggered acute exacerbations of IIPs and overall survival (OS) were analysed. The OS between groups of patients with and without the occurrence of acute exacerbation was compared at four landmark time points (30, 60, 90, and 120 days), starting from the first-line chemotherapy, using the landmark method. The incidence of acute exacerbation in patients who received first-line chemotherapy with small cell lung cancer (SCLC) and non-small cell lung cancer (NSCLC) was more frequent in NSCLC patients than in SCLC (4.2% vs 12.6%; odds ratio [OR]: 3.316; 95% confidence interval [CI] 1.25–8.8). Median survival time was 9.9 months (95% CI 9.2–10.7). Patients who experienced acute exacerbation had significant worse survival outcomes than those who did not at various time points (30 days, hazard ratio [HR]: 5.191, 95% CI 2.889–9.328; 60 days, HR: 2.351, 95% CI 1.104–5.009; 90 days, HR: 2.416, 95% CI 1.232–4.739; and 120 days, HR: 2.521, 95% CI 1.357–4.681). Acute exacerbation during first-line chemotherapy can predict poor survival.

Trial Registration number: UMIN000018227.

## Introduction

Lung cancer is a major comorbidity of idiopathic interstitial pneumonia (IIP)^1–3^. The development of lung cancer negatively impacts prognosis compared to idiopathic pulmonary fibrosis (IPF) alone^2,4–6^. Male gender^3,4^, smoking history^3,4^, comorbidities with emphysema^3^, impaired predicted forced vital capacity^3,4^, and age at the IPF diagnosis^7^ have been reported as significant risk factors for development of lung cancer in patients with IPF. The reported cumulative incidence of lung cancer development in patients with IPF is approximately 12.2–15.9% within five years and 23.3–31.1% within 10 years^3,4,7^. Lung cancer is sometimes difficult to identify on high-resolution computed tomography (HRCT) because of its atypical shape and unique tumour location adjacent to fibrotic lesions^8–10^. Not a small number of patients with lung cancer and IIPs receive a diagnosis at advanced stages. It has been observed that around 50% of patients who undergo surgical resection for lung cancer associated with IIP experience recurrence^11^.

The guideline-based therapy in patients with stage IV or post-operative recurrent disease is systemic chemotherapy. However, patients in IIP in this setting may not always receive systemic chemotherapy^5^, because the association of treatment efficacy of cytotoxic chemotherapy with advanced lung cancer and the risk of acute exacerbation of IIP remains inconclusive. Especially, acute exacerbation is a lethal complication typically occurring in patients with IPF provided approximately up to 50% mortality rate^12,13^. Acute exacerbation typically occurs in IPF patients. However, other fibrotic forms of IIPs also have the potential to cause acute exacerbation^14^. Most retrospective studies have included a small number of patients with some stage III locally advanced disease in addition to those of stage IV or post-operative recurrent disease^15,16^. In recent studies, various carboplatin-containing regimens have been investigated in a single-arm prospective manner^17–24^. It may be difficult to evaluate the risk of acute exacerbation because of the small number of patients in whom acute exacerbation was observed. There remains insufficient evidence regarding chemotherapy-related acute exacerbations, that is, triggered acute exacerbation^12^. A recent study from our group examining the use of chemotherapy in patients with advanced lung cancer and IIPs reported that administering chemotherapies to these patients improved survival outcomes and increased the risk of acute exacerbation compared to patients who received the best supportive care as an initial treatment^25^.

We conducted subgroup analyses using a chemotherapy group from our previous large retrospective multi-centre study^25^. A landmark analysis was performed to address whether patients who experience an acute exacerbation of IIPs during first-line chemotherapy might influence survival compared to patients who did not experience an acute exacerbation. In addition, predictors of poor survival and risk of chemotherapy-triggered acute exacerbation during first-line chemotherapy in real-world settings were explored.

## Materials and methods

### Study participants

We included subjects who met the following criteria; individuals who received chemotherapy as their initial treatment in our previous study^25^ and for whom data were available regarding the occurrence of acute exacerbation during first-line chemotherapy, the date of diagnosis with acute exacerbation, and the outcome. Considering these criteria, we aimed to ensure a focused and relevant sample for our research^25^.

### Study design

Following the amended Declaration of Helsinki, this retrospective multi-centre cohort study was conducted in 110 facilities. From each facility, we collected data from consecutive patients aged 20 years who were pathologically diagnosed with stage IV lung cancer or demonstrated post-operative recurrent disease from January 2012 to December 2013 and underwent chemotherapy or BSC as initial treatment^25^. The institutions that contributed to this study included academic medical centres and citizen hospitals belonging to the Japanese Respiratory Society (JRS). The protocol was approved by the local ethics committee of Toranomon Hospital (approval number: #1067) (ID: UMIN000018227). The committee of Toranomon Hospital waived the written informed consent requirement due to the study’s retrospective nature. Instead, a summary of the study protocol was posted on the hospital website in an opt-out format, allowing candidates the opportunity to express their desire not to participate in the study. The same protocol was applied and approved to the local committees of all collaborating facilities listed in acknowledgement section.

### Methods

We retrospectively reviewed the patient's medical records, including their demographic characteristics, as in our previous study using the data of chemotherapy group^25^. Radiological diagnoses were made based on HRCT patterns according to the international consensus guidelines provided by the American Thoracic Society/European Respiratory Society/JRS/Latin American Thoracic Association in 2011^26^. A lung cancer diagnosis was defined as the date of clinically confirmed stage IV or postoperative recurrence, in addition to the pathologic diagnosis. The Eastern Cooperative Oncology Group performance status (PS) system indicates the patients' general status^27^. Clinical data were collected during lung cancer diagnosis or as close to the diagnosis as possible.

As in our previous report displayed^25^, an acute exacerbation was defined based on the JRS guidelines^14^. The American Thoracic Society/European Respiratory Society^12^ proposed a set of criteria to identify triggers for acute exacerbations. Our study defined overall survival (OS) as the duration from the start of first-line chemotherapy to the time of death, which differed from our previous report^25^. We evaluated the initial treatment overall response and disease control rates (ORRs and DCRs) using the Response Evaluation Criteria in Solid Tumours (RECIST). The primary focus of this study was to investigate the impact of acute exacerbations on OS and the risks of chemotherapy-triggered acute exacerbation during first-line chemotherapy.

### Statistical analysis

We conducted a landmark analysis to compare OS between patients who developed acute exacerbation during the first-line chemotherapy period and those without exacerbation. This analysis was conducted at specific landmark time points at 30, 60, 90, and 120 days from the initiation of first-line chemotherapy. Landmark analysis is a valuable method for mitigating the potential bias known as ‘the guarantee-time bias’ when assessing OS between two groups of patients with and without acute exacerbation. To address this bias, patients who had died prior to each landmark time point were excluded from subsequent time point analyses. The remaining survivors at each successive landmark point were then used for the next survival analysis. The group of acute exacerbation (AE group) included survivors who experienced acute exacerbation until the date of each landmark point. Conversely, survivors who had not experienced acute exacerbation events up to each landmark date were included in the non-acute exacerbation group (non-AE group), even if some of them later experienced acute exacerbation events beyond each respective date. From each landmark time point, the survival of these two groups was compared using the Kaplan–Meier survival curve. Statistical differences in time-to-event outcomes were assessed using either the log-rank test or a Cox regression model.

For identifying individual risk factors associated with acute exacerbation and patient survival during first-line chemotherapy, univariate logistic and Cox regression models were employed, respectively. To address missing data within the entire cohort, a comprehensive dataset was constructed. Univariate analyses were performed on this complete dataset, which included cases with no missing clinical data, using logistic regression and Cox regression models. Additionally, multivariate logistic and Cox regression models were used to estimate the joint effects of risk factors on acute exacerbation and patient survival during first-line chemotherapy. In the multivariate analyses, only variables that were statistically significant in the univariate analysis were included.

All statistical analyses were performed using SAS version 9.4 (SAS Institute Inc.). Two-tailed *p*-values were reported. A *p-*value of < 0.05 was considered statistically significant.

## Results

**Figure 1:**
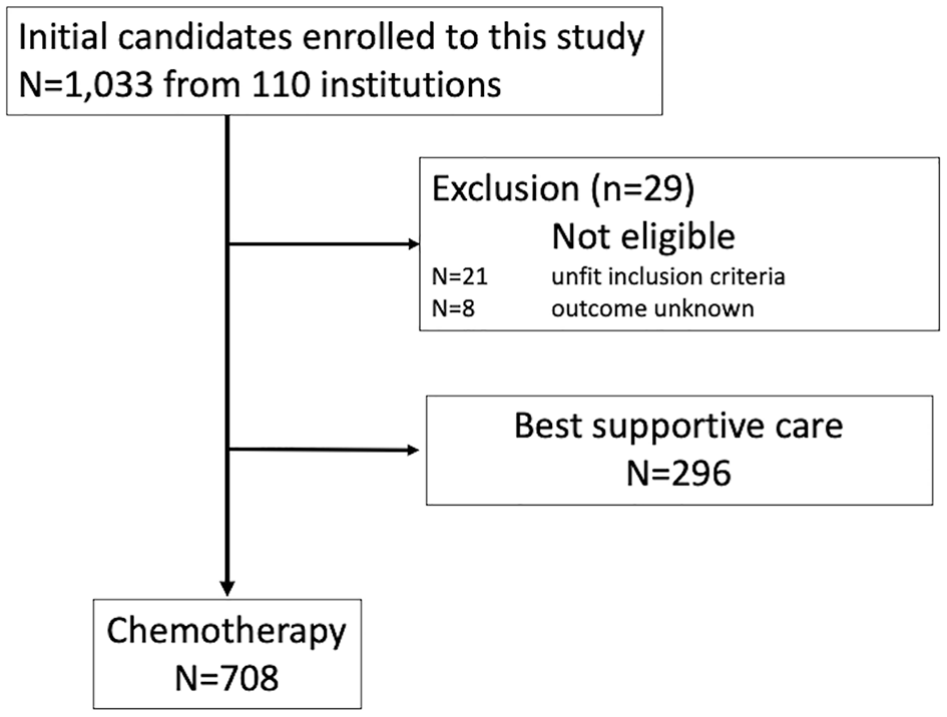
Flow chart for patients’ selection of this study.

The dataset comprised 708 cases (Fig. 1). Table 1 presents the patient characteristics. Table 2 lists the first-line chemotherapy regimens utilised in the study. Acute exacerbation during first-line chemotherapy was observed in 71 patients. Of 22 patients (31.0%) were died with median survival time with 2.14 [95% CI 1.05–2.73] months. Pulmonary function test results were relatively preserved with predicted forced vital capacity, but moderately impaired predicted diffusion capacity of the lung for carbon monoxide (%DLco). Nine patients were observed with %DLco less than 30%, and 59 patients were observed with %DLco of 30% or more but less than 50%.

**Table 1:** Patients’ demographics.

	Chemotherapy group
Participants (n)	708
Age (years)^#^	70.4 ± 6.9
Sex (male/female)	645/63
Smoking history (presence/absence/unknown)	684/20/4
Smoking index (pack-years)^#^	N = 677, 55.3 ± 30
Emphysema (presence/absence/unknown)	326/381/1
Performance status	
0/1/2/3/4	226/378/84/16/4
Interstitial pneumonia	
Clinical diagnosis of IIP (IPF/non-IPF/unknown)	406/294/8
HRCT pattern	
UIP pattern	275
Possible UIP pattern	243
Inconsistent with the UIP pattern	190
History of acute exacerbation (presence/absence/unknown)	11/689/8
Desaturation on exertion (presence/absence/unknown)	80/418/210
%FVC (%)^#^	n = 441, 88.6 ± 19.14
%DLco (%)^#^	n = 238, 64 ± 22.56
KL-6 (U/mL)^#^	n = 564, 922.8 ± 985.25
SP-D (ng/mL)^#^	n = 406, 135.6 ± 102.9
Treatment	
None	678
Prednisolone	15
Prednisolone + immunosuppressants	5
Pirfenidone	7
NAC	1
Pirfenidone + NAC	1
Pirfenidone + prednisolone	1
Lung cancer	
Histopathologic type	
Small cell carcinoma	216
Non-small cell carcinoma	492
Adenocarcinoma	258
Squamous cell carcinoma	173
LCNEC	17
LCC	9
Others	35
EGFR mutation status	
Negative	274
Positive	13
L858R/deletion 21	9/4
Not evaluated	421
ALK re-arrangement	
Negative	124
Positive	2
Not evaluated	582

*ALK* anaplastic lymphoma kinase, *BSC* best supportive care, *EGFR* epidermal growth factor receptor, %DLco percentage of predicted diffusing capacity of the lung for monoxide, %*FVC* percentage of predicted forced vital capacity, *HRCT* high-resolution computed tomography, *IIP* idiopathic interstitial pneumonia, *IPF* idiopathic pulmonary fibrosis, *KL*-6 Krebs von den Lungen-6, *LCC* large cell carcinoma, *LCNEC* large cell neuroendocrine carcinoma, *NAC* inhaled N-acetylcysteine, *PS* eastern cooperative oncology group performance status, *SP*-*D* surfactant protein-D, *UIP* usual interstitial pneumonia.

^#^Mean ± standard deviation.

**Table 2:** Chemotherapy regimens.

Regimen	n	ORR (%)	DCR (%)	AE (n)	AE incidence (%)
< NSCLC >					
Tri-weekly CBDCA + PTX	113	32.7	62.8	13	11.5
CBDCA + PEM	61	21.3	54.1	10	16.4
CBDCA + S-1	42	23.8	50.0	2	4.8
CBDCA + PTX + Bev	32	59.4	81.3	4	12.5
CDDP + PEM	26	38.5	57.7	3	11.5
DTX	26	11.5	34.6	9	34.6
CBDCA + nab-PTX	26	42.3	69.2	1	3.8
CBDCA + PEM + Bev	23	43.5	87.0	3	13.0
Weekly CBDCA + PTX	21	42.9	71.4	1	4.8
PEM	17	11.8	23.5	3	17.6
CDDP + DTX	15	60.0	80.0	2	13.3
S-1	13	0	38.5	2	15.4
VNR	12	8.3	33.3	3	25.0
CDDP + VNR	11	27.3	63.6	0	0
CBDCA + VP16	11	9.1	45.5	1	9.1
Others^#^	43	–	–	5	11.6
Total	492	29.3	58.7	62	12.6
< SCLC >					
CBDCA + VP16	160	51.2	64.4	5	3.1
CDDP + VP16	34	52.9	70.6	2	5.9
CDDP + CPT11	10	50.0	80.0	0	0
CBDCA + CPT11	6	66.7	66.7	1	16.7
Others^##^	6	–	–	1	16.7
Total	216	51.4	65.7	9	4.2

*AE* acute exacerbation, *AMR* amrubicin, *Bev* bevacizumab, *CBDCA* carboplatin, *CDDP* cisplatin, *CPT*-*11* irinotecan, *DTX* docetaxel, *NDP* nedaplatin, *nab*-*PTX* nanoparticle albumin-bound paclitaxel, *NGT* nogitecan, *NSCLC* non-small cell lung cancer, *ORR* objective response rate, *PEM* pemetrexed, *PTX* paclitaxel, *SCLC* small cell lung cancer, *VNR* vinorelbine; *VP*-16 etoposide.

^#^'Others' includes regimens consisting of 10 cases or less, i.e. CDDP + S-1 (n = 9), CDDP + VP-16 (n = 4), CBDCA + DTX (n = 3), CBDCA + gemcitabine (GEM) (n = 3), CBDCA + VNR (n = 3), weekly CBDCA + PTX + Bev (n = 2), CBDCA + CPT-11 (n = 1), CBDCA + PTX + Bev (n = 1), CDDP + GEM (n = 2), CDDP + S-1 + Bev (n = 1), PTX (n = 5), Gefitinib (n = 3), GEM (n = 2), Nedaplatin (n = 1), Bev (n = 1), UFT (n = 1), and PEM + BEV (n = 1).

^##^'Others' includes regimens consisting of five cases or less, i.e. AMR (n = 3), monthly CBDCA + PTX (n = 2), and VP-16 (n = 1).

### The first-line chemotherapy regimens used

In NSCLC patients, there were several treatment options available, and the ORR and DCR were estimated at 29.3% and 58.7%, respectively. Among SCLC patients, the most commonly used regimen was carboplatin (CBDCA) + etoposide (VP16) in 160 patients, which had a relatively low incidence rate of acute exacerbation (3.1%). In contrast to NSCLC regimens, most SCLC patients received CBDCA or cisplatin (CDDP) + VP16 (160/34; 89.8%), with a relatively homogeneous selection of regimens. The ORR and DCR in SCLC were 51.4% and 65.7%, respectively. Overall, the incidence of acute exacerbations was lower in SCLC (4.2%) than in NSCLC (12.6%).

Few patients (N = 13) whose epithelial growth factor receptor mutation status was positive were observed. Three of 13 patients received gefitinib as first-line chemotherapy, resulting that acute exacerbation occurred in 2 of three patients. Among other 10 patients, 8 received platinum doublet regimens (CBDCA + paclitaxel in three and CDDP + TS-1, CDDP + pemetrexed [PEM], CBDCA + PEM, CBDCA + PEM + Bevacizumab, CDDP + vinorelbine [VNR] in one each, respectively) and remaining two patients received DOC monotherapy. Acute exacerbation was occurred none of these 10 patients.

### Occurrence of acute exacerbation predicts poor survival

**Figure 2:**
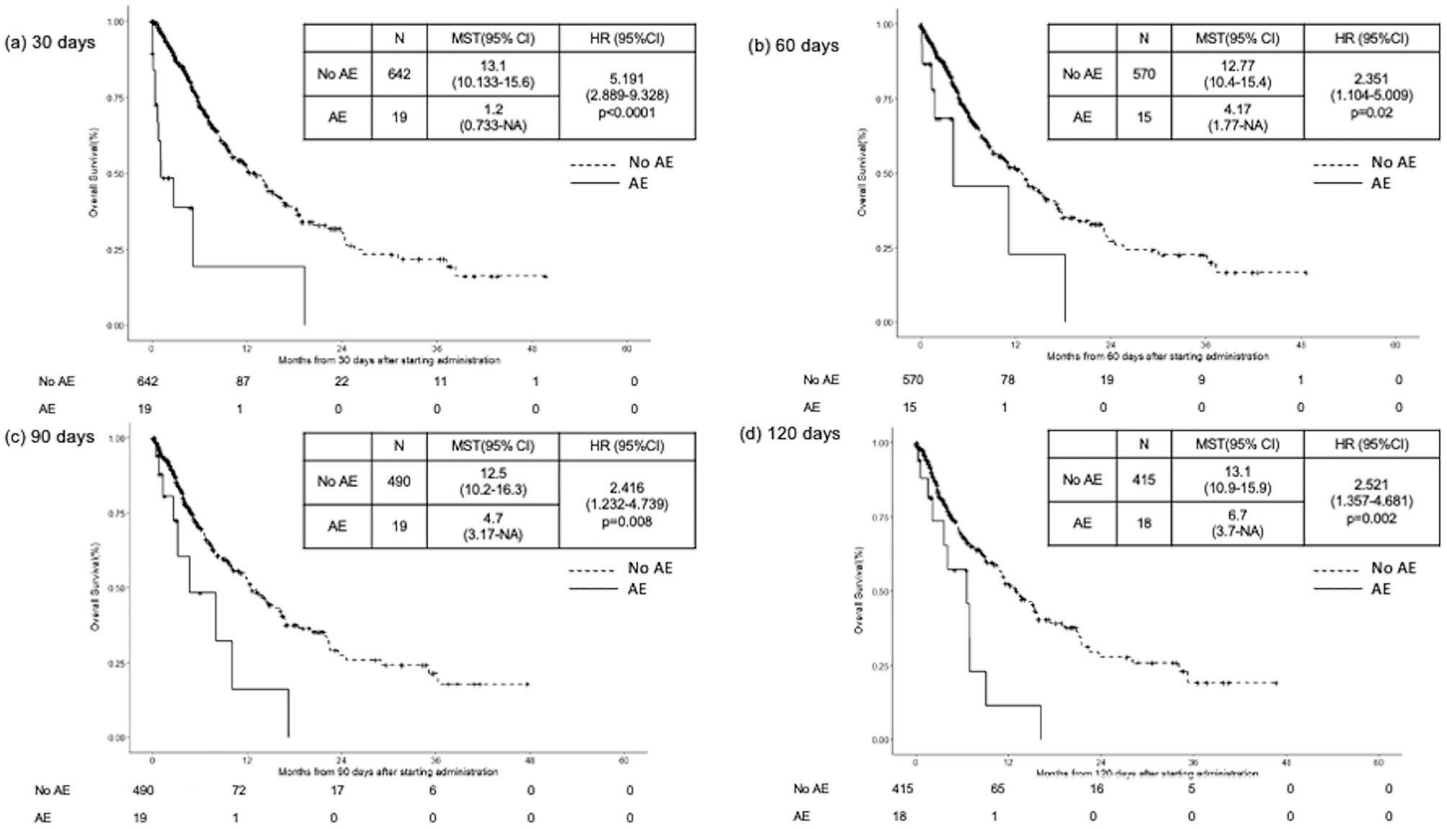
Landmark analysis. Kaplan–Meier survival curve for comparison of the group which experienced acute exacerbation (AE group) with one which did not experience acute exacerbation (no AE group) during the first-line chemotherapy with landmark points of (**a**) 30, (**b**) 60, (**c**) 90, and (**d**) 120 days, respectively, after the date of administration of the first-line regimen.

Landmark analysis indicated that patients who experienced acute exacerbation divided by landmark points (30, 60, 90, and 120 days from the date of initiation of first-line chemotherapy) had poorer OS than those who did not experience acute exacerbation (30 days, p < 0.0001; 60 days, p = 0.02; 90 days, p = 0.008; 120 days, p = 0.002) (Fig. 2).

**Figure 3:**
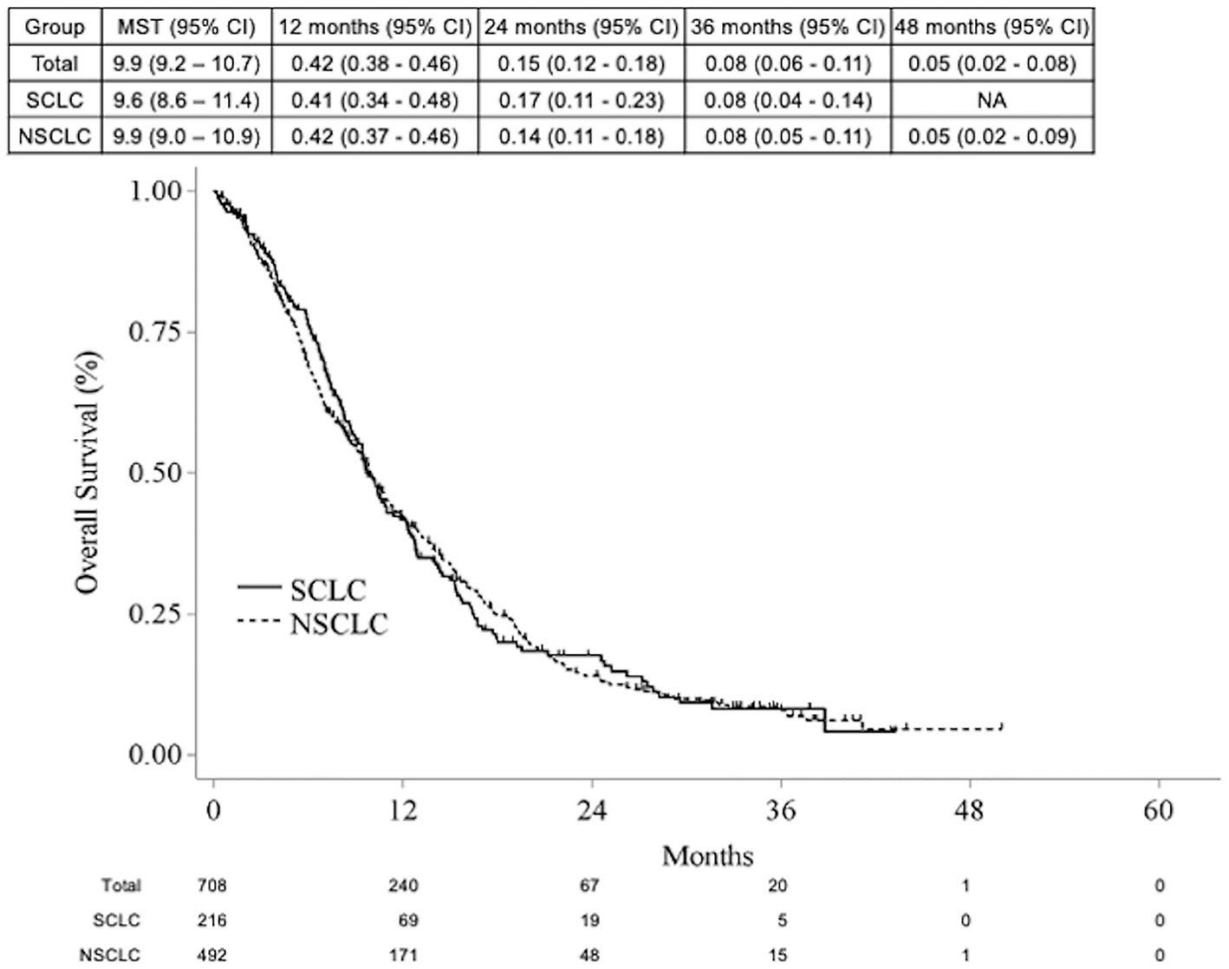
Kaplan–Meier survival curve for subgroups of NSCLC (n = 492) and SCLC (n = 216) with median survival time and 1, 2, 3 and 4 year-survival rates.

The OS for the entire cohort was 9.9 months (95% CI 9.2–10.7), while for patients with SCLC, it was 9.6 months (95% CI 8.6–11.4), and for those with NSCLC, it was 9.9 months (95% CI 9.0–10.9) (Fig. 3).

### Predictors of the risks of chemotherapy-triggered acute exacerbation and poor survival

The univariate analyses conducted using data from the entire cohort (n = 708) revealed that advanced age (≥ 70) (OR: 1.844, 95% CI 1.082–3.142, p = 0.0245) and the use of regimens specifically designed for NSCLC histology (OR: 3.316, 95% CI 1.617–6.803, p = 0.0011) were identified as potential predictors of acute exacerbation during first-line chemotherapy. In the analysis of the complete dataset (n = 397), the results of the univariate analyses demonstrated that regimens specifically designed for NSCLC were found to be statistically significant (OR: 3.258, 95% CI 1.249–8.498, p = 0.0158). In the multivariate logistic regression analysis, adjusted for age and gender, it was found that regimens specifically designed for NSCLC were an independent risk factor for predicting the occurrence of chemotherapy-triggered acute exacerbation during first-line chemotherapy (OR: 3.316, 95% CI 1.25–8.8, p = 0.016); this may indicate that the choice of NSCLC regimens significantly influenced the risk of acute exacerbation during the first-line treatment. These findings are summarised in Table 3.

**Table 3:** Univariate and multivariate analyses with a logistic regression model for the risk of acute exacerbation.

		Univariate (n = 708)				Univariate (N = 397)				Multivariate		
		N	OR	95% CI	P value	N	OR	95% CI	P value	OR	95% CI	P value
Sex	Female	63	Ref			39	Ref			Ref		
	Male	645	2.357	0.72–7.718	0.1576	358	2.458	0.572–10.572	0.2268	2.178	0.493–9.619	0.3042
Age	< 70	299	Ref			171	Ref			Ref		
	≧ 70	409	1.844	1.082–3.142	0.0245	226	1.531	0.793–2.955	0.2046	1.432	0.721–2.844	0.3051
PS	0	226	Ref			133	Ref			Ref		
	1	378	0.828	0.467–1.469	0.5197	212	1.05	0.496–2.226	0.898	0.945	0.435–2.053	0.8863
	≧ 2	104	1.941	0.991–3.8	0.0531	52	3.025	1.26–7.268	0.0133	2.603	0.992–6.83	0.052
Smoking index	< 50	345	Ref									
	≧ 50	352	1.01	0.614–1.66	0.9689							
Clinical diagnosis of IIPs	Not-IPF	294	Ref									
	IPF	406	0.817	0.499–1.337	0.4204							
HRCT pattern	Inconsistent with the UIP pattern	190	Ref									
	UIP pattern	243	0.975	0.524–1.814	0.9357							
	Possible UIP pattern	275	0.888	0.48–1.641	0.7037							
History of AE	Presence	11	Ref									
	Absence	689	–	–	0.9839							
Emphysema	Absence	381	Ref									
	Presence	326	0.788	0.479–1.297	0.3489							
KL-6	< 500	184	Ref			138	Ref			Ref		
	≧ 500, < 1000	221	1.373	0.697–2.701	0.3592	148	1.165	0.539–2.521	0.6974	1.04	0.466–2.322	0.9229
	≧ 1000, < 2000	124	1.55	0.729–3.299	0.255	87	1.539	0.668–3.547	0.3118	1.292	0.535–3.121	0.5686
	≧ 2000	35	1.878	0.635–5.552	0.2546	24	1.374	0.36–5.234	0.6419	1.258	0.317–4.985	0.7442
SP-D	< 110	203	Ref									
	≧ 110, < 150	71	1.794	0.78–4.125	0.1692							
	≧ 150, < 250	80	1.931	0.877–4.252	0.1024							
	≧ 250	52	2.29	0.956–5.485	0.063							
%FVC	≧ 80	296	Ref									
	≧ 50, < 80	137	1.014	0.53–1.943	0.9657							
	< 50	8	–	–	0.9859							
%DLco	≧ 80	55	Ref									
	< 80	183	1.656	0.604–4.541	0.327							
Desaturation on exertion	Absence	418	Ref			330	Ref			Ref		
	Presence	80	1.765	0.878–3.548	0.111	67	2.032	0.986–4.187	0.0547	1.416	0.622–3.224	0.4077
Treatment for IIPs	No	678	Ref									
	Yes	30	1.855	0.687–5.007	0.2229							
Histology of lung cancer	SCLC	216	Ref			109	Ref			Ref		
	NSCLC	492	3.316	1.617–6.803	0.0011	288	3.258	1.249–8.498	0.0158	3.316	1.25–8.8	0.016

*AE* acute exacerbation, *CI* confidence interval, %*Dlco* predicted diffusing capacity of the lung for monoxide, %*FVC* predicted forced vital capacity, *HRCT* high-resolution computed tomography, *Hx* histology, *IP* interstitial pneumonia, *KL*-*6* Krebs von den Lungen-6, *NS* not significant, *NSCLC* non-small cell lung cancer, *OR* odds ratio, *PaO2* partial arterial pressure of oxygen, *PS* performance status, *ref* reference, *SCLC* small cell lung cancer, *SP*-*D* surfactant protein-D, *UIP* usual interstitial pneumonia.

In the univariate analyses conducted on the entire cohort (n = 708), several factors were identified as potential predictors of poor survival. These included male sex (HR: 1.363, 95% CI 1.01–1.839, p = 0.0427), PS of 1 (HR: 1.546, 95% CI 1.273–1.876, p < 0.0001) and PS of ≥ 2 (HR: 3.331, 95% CI 2.548–4.355, p < 0.0001), higher serum levels of Krebs von den Lungen-6 (KL-6) (500≦KL-6 < 1000: HR: 1.305, 95% CI 1.04–1.638, p = 0.0216; 1000 ≦ KL-6 < 2000: HR: 1.441, 95% CI 1.105–1.879, p = 0.007), and the presence of desaturation on exertion (HR: 1.57, 95% CI 1.177–2.096, p = 0.0022). Using the complete dataset (n = 397), the results of the univariate analyses were almost the same as those of the entire cohort. The multivariate Cox regression analysis adjusted for age and sex demonstrated that poor PS of 1 (HR, 2.222; 95% CI 1.682–2.936; p < 0.0001) and ≥ 2 (HR: 4.006, 95% CI 2.627–6.108, p < 0.0001) was a significant predictor of death. These findings are summarised in Table 4.

**Table 4:** Univariate and multivariate analyses with the Cox regression hazard model for survival.

		Univariate (n = 708)				Univariate (N = 397)				Multivariate		
		N	HR	95% CI	P value	N	HR	95% CI	P value	HR	95% CI	P value
Sex	Female	63	Ref			39	Ref			Ref		
	Male	645	1.363	1.01–1.839	0.0427	358	1.213	0.83–1.772	0.3189	1.322	0.898–1.945	0.1569
Age	< 70	299	Ref			171	Ref			Ref		
	≧ 70	409	1.057	0.892–1.254	0.5217	226	0.941	0.745–1.19	0.6131	0.937	0.739–1.189	0.5919
PS	0	226	Ref			133	Ref					
	1	378	1.546	1.273–1.876	< 0.0001	212	2.203	1.674–2.899	< 0.0001	2.222	1.682–2.936	< 0.0001
	≧ 2	104	3.331	2.548–4.355	< 0.0001	52	4.142	2.822–6.08	< 0.0001	4.006	2.627–6.108	< 0.0001
Smoking index	< 50	345	Ref									
	≧ 50	352	1.039	0.876–1.233	0.6581							
Clinical diagnosis of IIPs	Not-IPF	294	Ref									
	IPF	406	1.155	0.972–1.373	0.1022							
HRCT pattern	Inconsistent with the UIP pattern	190	Ref									
	UIP pattern	243	0.884	0.712–1.098	0.265							
	Possible UIP pattern	275	1.095	0.888–1.349	0.3973							
History of AE	Presence	11	Ref									
	Absence	689	1.45	0.775–2.714	0.2445							
Emphysema	Absence	381	Ref									
	Presence	326	1.109	0.936–1.315	0.2317							
KL-6	< 500	184	Ref			138	Ref			Ref		
	≧500, < 1000	221	1.305	1.04–1.638	0.0216	148	1.312	0.997–1.726	0.0522	1.161	0.871–1.547	0.308
	≧1000, < 2000	124	1.441	1.105–1.879	0.007	87	1.425	1.035–1.96	0.0298	1.323	0.952–1.841	0.0959
	≧2000	35	1.295	0.85–1.974	0.2292	24	1.367	0.804–2.323	0.2484	1.379	0.804–2.363	0.2428
SP-D	< 110	203	Ref									
	≧ 110, < 150	71	0.848	0.627–1.149	0.2882							
	≧ 150, < 250	80	1.128	0.835–1.524	0.433							
	≧ 250	52	0.862	0.604–1.231	0.4147							
%FVC	≧ 80	296	Ref									
	≧ 50, < 80	137	1.219	0.959–1.549	0.1053							
	< 50	8	1.64	0.674–3.99	0.2759							
%DLco	≧ 80	55	Ref									
	< 80	183	1.172	0.826–1.664	0.3726							
Desaturation on exertion	Absence	418	Ref			330	Ref			Ref		
	Presence	80	1.57	1.177–2.096	0.0022	67	1.663	1.209–2.288	0.0018	0.858	0.654–1.125	0.2681
Treatment for IIPs	No	678	Ref									
	Yes	30	1.11	0.724–1.702	0.6309							
Histology of lung cancer	SCLC	216	Ref			109	Ref			Ref		
	NSCLC	492	1.01	0.839–1.217	0.9134	288	0.962	0.738–1.254	0.7729	1.094	0.767–1.561	0.6191

*AE* acute exacerbation, *CI* confidence interval, %*Dlco* predicted diffusing capacity of the lung for monoxide, %*FVC* predicted forced vital capacity, *HRCT* high-resolution computed tomography, *Hx* histology, *IP* interstitial pneumonia, *KL*-*6* Krebs von den Lungen-6, *NS* not significant, *NSCLC* non-small cell lung cancer, *OR* odds ratio, *PaO2* partial arterial pressure of oxygen, *PS* performance status, *ref* reference, *SCLC* small cell lung cancer, *SP*-*D* surfactant protein-D, *UIP* usual interstitial pneumonia.

## Discussion

The current study demonstrated that patients who experienced acute exacerbations during first-line chemotherapy had significantly worse survival rates than those who did not. Poor PS was significantly associated with poor survival. Compared with SCLC, some NSCLC regimens may potentially lead to acute exacerbation of IIP in patients who received first-line chemotherapy.

No appropriate first-line chemotherapy has been established for IIP patients with advanced NSCLC or SCLC. Several prospective, small-sized, single-arm studies have assessed the validity and/or feasibility of CBDCA + weekly paclitaxel (PTX)^22,23^, CBDA + S-1^18,19^, and CBDCA + nab-PTX^20,21,24^ in patients with NSCLC and interstitial lung disease (ILD). These studies reported the following findings: OS ranged from 9.7 to 19.8 months, ORR ranged from 33.3 to 69.7%, DCR ranged from 66.7 to 93.9%, and the occurrence rates of acute exacerbation ranged from 4.3 to 12.1%. Otsubo et al.^28^ prospectively investigated the acute exacerbation rate by comparing patients with IPF and advanced lung cancer who received CBDCA + nab-PTX with and without nintedanib. No statistical difference was observed in the acute exacerbation rate between the groups (event free survival: 14.6 vs. 11.8 months). Furthermore, for patients with extensive SCLC and ILD, there is scarce evidence for suitable and established first-line chemotherapy. One prospective study has used CBDCA + VP-16 in lung cancer treatment. Reported outcomes include OS (8.7 months) and acute exacerbation occurrence rates (5.8%)^17^. Minegishi et al.^15^ conducted a large retrospective multi-centre cohort study with 204 NSCLC and 74 SCLC patients. This study presented the OS of patients with NSCLC and SCLC at 14.3 and eight months after first-line chemotherapy, respectively. Patient demographics in these studies, including lung cancer stage (NSCLC: III/IV or SCLC: limited/extensive), pulmonary function status, ILD clinical diagnosis (IPF vs non-IPF), HRCT findings (UIP pattern or others), and the study period for acute exacerbation development (chemotherapy period only or inclusive of best supportive care period), exhibited considerable variability. Due to this diversity, these results remain inconclusive for even the occurrence rate of acute exacerbations in this population. The results of the present study correspond to real-world clinical settings during first-line chemotherapy. Notably, the stage of lung cancer was restricted to stage IV or postoperative recurrent disease in this study.

Landmark analysis has played a crucial role in addressing the clinical question regarding the prognostic implications of acute exacerbation during first-line chemotherapy compared with that noted in patients without such events. Consistently, our findings indicate that patients who experienced acute exacerbation during their first-line chemotherapy had poorer survival outcomes than those who did not, highlighting the significance of acute exacerbation as a prognostic factor in lung cancer treatment. Moreover, our previous study has shown that chemotherapy can predict the occurrence of acute exacerbation compared with that noted with the best supportive care^25^. Based on this prediction, patients who experience acute exacerbation during first-line chemotherapy might be reasonably advised to transition their treatment strategies to the best supportive care rather than further continuation of chemotherapy, whereas it may be difficult to design further studies to directly compare OS experiencing an acute exacerbation on chemotherapy versus best supportive care. In contrast, our previous study demonstrated that first-line chemotherapy provided a survival benefit compared with that noted when choosing the best supportive care as the initial treatment^25^. Appropriate clinical follow-ups and evaluations are required during chemotherapy to confirm the occurrence of acute exacerbations. However, based on our current analyses, it remains unclear which patients have the potential to develop acute exacerbation.

Notably, NSCLC patients were identified as having a significantly higher risk of acute exacerbation during first-line chemotherapy than patients with SCLC, although this result should be interpreted with caution. Impaired lung function (lower predicted forced vital capacity)^29–31^, histologic type of NSCLC^29^, age < 70 years^32^, poor PS (2, 3)^32^, and UIP pattern on HRCT^32^ have been reported as risk factors for chemotherapy-triggered acute exacerbations, as analysed by a logistic regression method. Whether the histopathological type itself could affect the occurrence of acute exacerbation remains to be elucidated. One possible interpretation may be that the relatively homogeneous usage of chemotherapy regimens with CBDCA/CDDP + VP16 in SCLC patients and other extremely varied regimens (N = 16) for NSCLC patients might affect the result. CBDCA/CDDP + VP16 was reported as the regimen that may have relatively low occurrence rate of acute exacerbation^15^. Conversely, some specific NSCLC regimens may indicate a high potential for acute exacerbation. The potential risk of a relatively high occurrence of acute exacerbation with specific regimens, including PEM or DOC monotherapy, has been discussed^33–35^. The current study could not determine which regimens were safer. In addition, in the previous study^25^, chemotherapy predicted better survival than best supportive care in any subgroup analyses. Overall, the histological type of NSCLC itself does not negatively impact the initiation of chemotherapy in these patients. However, further studies are required to resolve this issue.

This study confirms that poor PS is a significant predictor of survival; this finding is consistent with previous studies based on SCLC and NSCLC^33,36,37^. PS is pivotal in treatment decision-making and evaluating patient outcomes. In addition, elevated serum lactate dehydrogenase levels^38^ and C-reactive protein levels^37^, along with a clinical diagnosis of IPF^33,38,39^, have been identified as detrimental factors associated with poorer outcomes in SCLC or NSCLC. Importantly, these studies evaluated poor predictors of survival using a cohort that included patients with and without pre-existing ILD^33,37–39^. This study identified poor PS as the sole independent predictor of poor outcomes. Interestingly, none of the other variables examined were found to be significant predictors. It is noteworthy that this study evaluated a large cohort of patients with stage IV or post-operated disease with ILD only, providing valuable insights into the predictive factors associated with adverse prognosis in this population. However, nothing can be concluded to date because no direct comparison of IIP and lung cancer with IIP alone was performed in the current study. Further studies are required to confirm this hypothesis.

This study had several limitations. First, this study was retrospective nature. Therefore, some defects in the clinical information regarding IIPs, lung cancer, and outcomes were included. Missing information made it difficult to achieve perfect results from the multivariate analyses. Second, due to the small numbers and various regimens used, assessing each regimen's comparative therapeutic benefits, including OS and PFS was challenging. In addition, it remains inconclusive whether adverse events other than acute exacerbation might influence the outcome because they were not collected due to per-protocol issues. Third, the participants in this study were recruited from January 2012 to December 2013 before an era in which immune checkpoint inhibitors were available. Because this study included the data regarding the patients who treat purely cytotoxic agents, this data would be valuable reference when further studies will be performed to investigate the additional efficacy and risk of combination regimens with cytotoxic agent plus immune checkpoint inhibitors in this population, thereafter one retrospective study was recently published in patients who treated with immune checkpoint inhibitor^40^. In addition, some prospectively assessed regimens (CBDCA + weekly paclitaxel (PTX)^22,23^, CBDA + S-1^18,19^, and CBDCA + nab-PTX^20,21,24^ in patients with NSCLC) are possible candidates to date. However, at an enrolment, regimen selection was exploratory. The reason why various regimens were identified in NSCLC may be that the participants enrolment was before an era in which these prospective studies were actively published. Fourth, small number of patients (N = 14) who received antifibrotic therapy was identified in this study. This may also associate with the study period. Pirfenidone has been available since 2008 in Japan, however, nintedanib was not yet available. Pirfenidone was majorly prescribed in patients with IPF alone with severe disease due to medical insurance issues at that time. As J-SONIC study investigated the value to add-on nintedanib to cytotoxic agents^28^, even recently, usage of antifibrotic agents in patients with interstitial pneumonia and advanced stage of lung cancer may be challenging^15^. Fifth, male predominance was shown in this study. Although it may be a common epidemiologic characteristic in previous studies^2,3^, unequal gender distribution may be a bias for outcome analyses.

In conclusion, if acute exacerbation occurs during first-line chemotherapy, switching to the best supportive care instead of continuing chemotherapy may be a possible option. Despite the overall clinical benefit of chemotherapy in terms of OS, the decision should be made with caution whether second line chemotherapy should be subsequently performed. Further studies will be necessary to confirm safety of second line chemotherapy.

Notably, the lower acute exacerbation rate in patients with SCLC may support the safer use of CBDCA/CDDP + VP16. While the risk of exacerbation of IIP may be higher with certain regimens for NSCLC than with SCLC, these findings should not discourage the use of chemotherapy in patients with NSCLC and IIPs who have a good PS.

## Data Availability

All de-identified data that underlie the reported results of this study are available from a corresponding author on reasonable request.

## Abbreviations

CI: Confidence interval
DLco: Diffusing capacity of the lung for monoxide
ERS: European respiratory society
HR: Hazard ratio
HRCT: High resolution computed tomography
IIP: Idiopathic interstitial pneumonia
IPF: Idiopathic pulmonary fibrosis
JRS: Japanese respiratory society
KL-6: Krebs von den Lungen-6
MST: Median survival time
NSCLC: Non-small cell lung cancer
OR: Odds ratio
OS: Overall survival
PaO_2_: Partial pressure of arterial oxygen
PS: Eastern cooperative oncology group performance status
SCLC: Small cell lung cancer
SMD: Standardized mean difference
SP-D: Surfactant protein-D
UIP: Usual interstitial pneumonia

## References

[1] Matsushita H, Tanaka S, Saiki Y (1995). Lung cancer associated with usual interstitial pneumonia. Pathol. Int..

[2] Tomassetti S, Gurioli C, Ryu JH (2015). The impact of lung cancer on survival of idiopathic pulmonary fibrosis. Chest.

[3] Kato E, Takayanagi N, Takaku Y (2018). Incidence and predictive factors of lung cancer in patients with idiopathic pulmonary fibrosis. ERJ Open Res..

[4] Yoo H, Jeong BH, Chung MJ (2019). Risk factors and clinical characteristics of lung cancer in idiopathic pulmonary fibrosis: A retrospective cohort study. BMC Pulm. Med..

[5] Brown SW, Dobelle M, Padilla M (2019). Idiopathic pulmonary fibrosis and lung cancer. A systematic review and meta-analysis. Ann. Am. Thorac. Soc..

[6] Kim HC, Lee S, Song JW (2021). Impact of idiopathic pulmonary fibrosis on clinical outcomes of lung cancer patients. Sci. Rep..

[7] Song MJ, Kim SY, Park MS (2021). A nationwide population-based study of incidence and mortality of lung cancer in idiopathic pulmonary fibrosis. Sci. Rep..

[8] Yoshida R, Arakawa H, Kaji Y (2012). Lung cancer in chronic interstitial pneumonia: Early manifestation from serial CT observations. AJR Am. J. Roentgenol..

[9] Miyamoto A, Kurosaki A, Fujii T (2017). HRCT features of surgically resected invasive mucinous adenocarcinoma associated with interstitial pneumonia. Respirology.

[10] Miyamoto A, Kurosaki A, Moriguchi S (2019). Reduced area of the normal lung on high-resolution computed tomography predicts poor survival in patients with lung cancer and combined pulmonary fibrosis and emphysema. Respir. Investig..

[11] Sato T, Watanabe A, Kondo H (2015). Long-term results and predictors of survival after surgical resection of patients with lung cancer and interstitial lung diseases. J. Thorac Cardiovasc. Surg..

[12] Collard HR, Ryerson CJ, Corte TJ (2016). Acute exacerbation of idiopathic pulmonary fibrosis. An international working group report. Am. J. Respir. Crit. Care Med..

[13] Kondoh Y, Taniguchi H, Ebina M (2015). Risk factors for acute exacerbation of idiopathic pulmonary fibrosis–Extended analysis of pirfenidone trial in Japan. Respir. Investig..

[14] Suzuki A, Kondoh Y, Brown KK (2020). Acute exacerbations of fibrotic interstitial lung diseases. Respirology.

[15] Minegishi Y, Gemma A, Homma S (2020). Acute exacerbation of idiopathic interstitial pneumonias related to chemotherapy for lung cancer: Nationwide surveillance in Japan. ERJ Open Res..

[16] Wang Y, Miao L, Hu Y (2020). The efficacy and safety of first-line chemotherapy in patients with non-small cell lung cancer and interstitial lung disease: A systematic review and meta-analysis. Front. Oncol..

[17] Minegishi Y, Kuribayashi H, Kitamura K (2011). The feasibility study of carboplatin plus etoposide for advanced small cell lung cancer with idiopathic interstitial pneumonias. J. Thorac. Oncol..

[18] Sekine A, Satoh H, Baba T (2016). Safety and efficacy of S-1 in combination with carboplatin in non-small cell lung cancer patients with interstitial lung disease: A pilot study. Cancer Chemother. Pharmacol..

[19] Hanibuchi M, Kakiuchi S, Atagi S (2018). A multicenter, open-label, phase II trial of S-1 plus carboplatin in advanced non-small cell lung cancer patients with interstitial lung disease. Lung Cancer.

[20] Kenmotsu H, Yoh K, Mori K (2019). Phase II study of nab-paclitaxel + carboplatin for patients with non-small-cell lung cancer and interstitial lung disease. Cancer Sci..

[21] Asahina H, Oizumi S, Takamura K (2019). A prospective phase II study of carboplatin and nab-paclitaxel in patients with advanced non-small cell lung cancer and concomitant interstitial lung disease (HOT1302). Lung Cancer.

[22] Fukuizumi A, Minegishi Y, Omori M (2019). Weekly paclitaxel in combination with carboplatin for advanced non-small-cell lung cancer complicated by idiopathic interstitial pneumonias: A single-arm phase II study. Int. J. Clin. Oncol..

[23] Minegishi Y, Sudoh J, Kuribayasi H (2011). The safety and efficacy of weekly paclitaxel in combination with carboplatin for advanced non-small cell lung cancer with idiopathic interstitial pneumonias. Lung Cancer.

[24] Sakashita H, Uchibori K, Jin Y (2022). A phase II feasibility study of carboplatin and nab-paclitaxel for advanced non-small cell lung cancer patients with interstitial lung disease (YLOG0114). Thorac. Cancer.

[25] Miyamoto A, Michimae H, Nakahara Y (2023). Chemotherapy versus best supportive care in advanced lung cancer and idiopathic interstitial pneumonias: A retrospective multi-centre cohort study. Respir. Investig..

[26] Raghu G, Collard HR, Egan JJ (2011). An official ATS/ERS/JRS/ALAT statement: idiopathic pulmonary fibrosis: Evidence-based guidelines for diagnosis and management. Am. J. Respir. Crit. Care Med..

[27] Oken MM, Creech RH, Tormey DC (1982). Toxicity and response criteria of the Eastern Cooperative Oncology Group. Am. J. Clin. Oncol..

[28] Otsubo K, Kishimoto J, Ando M (2022). Nintedanib plus chemotherapy for non-small cell lung cancer with IPF: a randomized phase 3 trial. Eur. Respir. J..

[29] Enomoto Y, Inui N, Kato T (2016). Low forced vital capacity predicts cytotoxic chemotherapy-associated acute exacerbation of interstitial lung disease in patients with lung cancer. Lung Cancer.

[30] Kobayashi H, Omori S, Nakashima K (2017). Modified GAP index for prediction of acute exacerbation of idiopathic pulmonary fibrosis in non-small cell lung cancer. Respirology.

[31] Taya T, Chiba H, Yamada G (2019). Risk factors for acute exacerbation of idiopathic interstitial pneumonia in patients undergoing lung cancer treatment. Jpn. J. Clin. Oncol..

[32] Kenmotsu H, Naito T, Kimura M (2011). The risk of cytotoxic chemotherapy-related exacerbation of interstitial lung disease with lung cancer. J. Thorac. Oncol..

[33] Kanaji N, Tadokoro A, Kita N (2016). Impact of idiopathic pulmonary fibrosis on advanced non-small cell lung cancer survival. J. Cancer Res. Clin. Oncol..

[34] Kato M, Shukuya T, Takahashi F (2014). Pemetrexed for advanced non-small cell lung cancer patients with interstitial lung disease. BMC Cancer.

[35] Tamiya A, Naito T, Miura S (2012). Interstitial lung disease associated with docetaxel in patients with advanced non-small cell lung cancer. Anticancer Res..

[36] Nakao S, Yamaguchi K, Sakamoto S (2019). Chemotherapy-associated acute exacerbation of interstitial lung disease shortens survival especially in small cell lung cancer. Anticancer Res..

[37] Kashiwabara K, Semba H, Fujii S (2015). Difference in benefit of chemotherapy between small cell lung cancer patients with interstitial pneumonia and patients with non-small cell lung cancer. Anticancer Res..

[38] Koyama N, Iwai Y, Nagai Y (2019). Idiopathic pulmonary fibrosis in small cell lung cancer as a predictive factor for poor clinical outcome and risk of its exacerbation. PLoS One.

[39] Kanaji N, Shimizu J, Sakai K (2020). Clinical features of patients with small cell lung cancer and idiopathic pulmonary fibrosis treated with chemotherapy or chemoradiotherapy. Ther. Adv. Respir. Dis..

[40] Isobe K, Nakamura Y, Sakamoto S (2024). Immune checkpoint inhibitors in patients with lung cancer having chronic interstitial pneumonia. ERJ Open Res..

